# Prevalence of Vitamin D Deficiency and Associated Risk Factors in Cerebral Palsy A study in North-West of Iran 

**Published:** 2018

**Authors:** Vahideh TOOPCHIZADEH, Mohammad BARZEGAR, Shahab MASOUMI, Fatemeh JAHANJOO

**Affiliations:** 1 Physical Medicine and Rehabilitation Research Center, Tabriz University of Medical Sciences, Tabriz, Iran.; 2 Pediatric Health Research Center, Tabriz University of Medical Sciences, Tabriz, Iran.

**Keywords:** Cerebral Palsy, Children, 25-hydroxyvitamin D, Motor function

## Abstract

**Objective:**

This study aimed to compare the prevalence of 25-hydroxyvitamin D deficiency in cerebral palsied (CP) with healthy control children and evaluate possible correlations between 25-hydroxyvitamin D and severity of CP and motor function.

**Materials & Methods:**

In this case-control study, serum levels of 25-hydroxyvitamin D were evaluated in 65 children with CP and compared with 65 healthy children referred to Tabriz Pediatric Hospital, Tabriz, northwestern Iran in 2015. Blood samples were taken to measure levels of 25-hydroxyvitamin D, calcium, phosphorus and alkaline phosphatase. Regarding 25-hydroxyvitamin D levels, patients were classified as sufficient (≥30 ng/ml), insufficient (20-30 ng/ml) and deficient (<20 ng/ml).

**Results:**

Mean 25-hydroxyvitamin D levels were 28.03±24.2 ng/ml in patients and 30±1.94 ng/ml in control group. 25-hydroxyvitamin D deficiency was seen in 44.6% of CP and 18.5% of healthy children. There was no significant difference in 25-hydroxyvitamin D levels between boys and girls, CP types and use of antiepileptics in case group. There was significant negative correlation between age and 25-hydroxyvitamin D levels (*P*=0.007). The correlation between 25-hydroxyvitamin D and Gross Motor Function Classification System was not significant.

**Conclusion:**

25-hydroxyvitamin D deficiency is common in children with CP in comparison with healthy children. There was significant negative correlation between age and 25-hydroxyvitamin D levels. Routine measurement of 25-hydroxyvitamin D levels and its proper treatment is recommended to prevent its deficiency and subsequent consequences.

## Introduction

Cerebral palsy (CP) is the most common developmental disabilities in childhood which persist throughout the lifespan, is a clinical syndrome characterized by a persistent disorder in motor control and posture, and results from injury or dysfunction of the brain which is non-progressive (1, 2). The motor disorders of cerebral palsy are often accompanied by disturbances of sensation, cognition, communication and behavior, epilepsy, and secondary musculoskeletal problems (3, 4). Children with CP experience limitations in their daily activities e.g. feeding, dressing, bathing, and mobility due to abnormal muscle tone, involuntary movement, unsteady gait, problems with balance, and poor social functioning (5, 6). They have also difficulties in swallowing. Feeding difficulty and malnutrition are two of the associated problems in cerebral palsy (7). These patients usually have poor overall nutrition and insufficient calcium and 25-hydroxyvitamin D intake (8).

In childhood, 25-hydroxyvitamin D is crucial for bone growth, mineralization, and musculoskeletal health because it promotes the assimilation of nutritional calcium and phosphate. It also regulated numerous cellular functions and would play an important role in the risk of metabolic syndrome, diabetes, autoimmune diseases, and some types of cancer (9). “Severe 25-hydroxyvitamin D deficiency (VDD) can result in rickets, metabolic bone disease, and hypocalcemia during infant and childhood growth” (10).

Because of nutritional deficiency, lack of sun exposure and sedentary behavior, osteopenia and rickets are common in children with CP. Patients have significantly decreased bone mineral density. In addition, painful fractures with minor traumas are common (11). 

In this study, we aimed to assess prevalence of 25-hydroxyvitamin D deficiency in children with CP and possible correlations between 25-hydroxyvitamin D and severity of CP and motor function was evaluated. 

## Materials & Methods

In this case-control study, 25-hydroxyvitamin D status of 65 children with CP visiting outpatient clinics of Tabriz Pediatric Hospital, Tabriz, northwestern Iran in 2015 was evaluated and compared with healthy age and sex-matched children without history of chronic disease. Considering 95% confidence, power of 80% and according to the similar studies d=3.76, s_1_=8.75 and s_2_=6.24 we determined the sample size of 65 participants for each study group. Inclusion criteria were children of both sexes between 3-11 yr old with confirmed CP for case group and without any chronic disease for control group and no history of 25-hydroxyvitamin D or calcium supplement use in recent 2 months.

Ethics Committee of Tabriz University of Medical Sciences approved the study, and the written informed consent was obtained from the parents or legal guardians of children.

Demographic findings, history of supplement use, anti-epileptic medications use and Gross Motor Function Classification System (GMFCS) were recorded. Calcium, phosphorus, alkaline phosphatase and 25-hydroxyvitamin D levels were measured. 

A peripheral non-fasting venous blood sample was obtained for analysis of multiple factors relating to nutrition or bone metabolism. The hospital laboratory at the respective institutions determined serum levels of calcium, phosphate, alkaline phosphatase using Pars Azmoon DGKC spectrophotometry kits in case group. Serum 25-hydroxyvitamin D was measured in the Sheikh Al-Rais Laboratory using Eliza Diasource kits (Louvain-la-Neuve, Belgium) in both study groups. All evaluations were done in the same season (spring and summer). The values for 25-hydroxyvitamin D levels ≥30 ng/ml were considered as sufficient, between 20 and 30 ng/ml as insufficient and <20 ng/ml as deficient (12).

**GMFCS**

GMFCS is a standard observational tool for assessing children with cerebral palsy that evaluated the ability to perform movements such as walking, climbing stairs, running and sitting. According to this scale, children are placed into five grades from I to V in order to their gross motor skills and lower levels represent better gross motor skills, with I as the lightest and V as the most severe level (13). 

**Statistical Analysis**

All statistical tests were performed using SPSS for Windows Ver. 17 (Chicago, IL, USA). Quantitative data were presented as mean ± standard of error (S.E), while qualitative data were demonstrated as frequencies and percentages (percentage). After determining of frequency distribution of variables using Kolmogorov-Smirnov test, independent *t*-test, one-way and two-way ANOVA, chi-square test or Fisher’s exact tests and Pearson correlation test, as appropriate were used to compare data between groups of patients. Pearson correlation was used to evaluate the correlation between variables. A *P*-value of <0.05 was considered statistically significant. 

This study was approved by the Ethics Committee of Tabriz University of Medical Sciences and informed consents were obtained from all study participants. The registry number of confirmation letter was 5/4/2767 and ethical code of this study was 91/1-7/16. The measurement of serum levels of vitamin D were paid by the Research Center of Physical Medicine and Rehabilitation**.**

## Results

Sixty-five children with CP were evaluated for 25-hydroxyvitamin D and compared with 65 healthy children. Table 1 demonstrates demographic and laboratory findings between groups. There was no significant difference in age or gender between groups. 44.6% of cerebral palsied and 18.5% of control children had deficient levels of 25-hydroxyvitamin D, including 9 patients (13.8%) with severe deficiency (25-hydroxyvitamin D levels <10 ng/ml) in case group. Using post-hoc analysis showed that children with CP compared to normal group had significantly higher rate of vitamin D deficiency (*P*=0.008). Correlation between 25-hydroxyvitamin D and age was evaluated and there was significantly negative correlation between 25-hydroxyvitamin D and age (*P*=0.007). Using Two-way ANOVA, there was not a significant interaction between the effects of gender and groups on 25-hydroxyvitamin D levels (Table 1). Although main effect analysis did not show statistically significant differences, boy’s 25-hydroxyvitamin D levels were considerably higher in case group. 

Patients were mostly spastic (73.8%) and quadriplegic type (43.1%). Antiepileptic medication was positive in 19 patients in case group (with phenobarbital). Most patients had GMFCS level II and III. The result from exact test indicated that prevalence of 25-hydroxyvitamin D Deficiency was the same between GMFC categories (GMFCI- II- III vs. IV-V). Lower calcium levels (<8.5 µg/dL) were observed only in one patient (15%). Elevated levels of alkaline phosphatase and high serum phosphorus (>4.5 µg/dL) were observed in 29 (44.61%) and 22 (33.84%), respectively. None of the cases had lower serum levels of phosphorus. 

We found no significant difference between boys and girls, CP types and use of antiepileptics regarding 25-hydroxyvitamin D levels (Table 2). Correlation between 25-hydroxyvitamin D with GMFCS level, age, and laboratory findings was evaluated and there was only significantly negative correlation between 25-hydroxyvitamin D and age (r=-0.333, *P*=0.007). 

## Discussion

We evaluated the 25-hydroxyvitamin D levels in cerebral palsied and healthy children and observed higher rate of 25-hydroxyvitamin D deficiency (44.6%) and severe deficiency (13.8%) in CP cases. 

There is different reported prevalence of 25-hydroxyvitamin D deficiency around the world varying from 40% to 52.4% (14, 15). Among Iranian normal school-aged children and adolescents, two studies have reported high prevalence rate (16, 17). Children with CP have higher rate of 25-hydroxyvitamin D deficiency compared to normal children. However, in our study, it was similar to studies in other countries (14,15). The higher rate of 25-hydroxyvitamin D deficiency among children is suggested to be related to insufficient sun exposure, low physical activity, advancing age and pubertal stage (14-17). Recommended mechanisms among children with CP are poor nutritional status, oral motor dysfunction, feeding problems, insufficient calcium intake, non-ambulatory status, and anticonvulsant use (18). Antiepileptic drugs are associated with reduced 25-hydroxyvitamin D levels, rickets, and osteomalacia (19, 20). Anti-epileptic drugs, especially enzyme-inducing ones, may result in accelerated vitamin D metabolism and so vitamin D levels are decreased and consequently result in bone mineral density reduction (21). However, we found no significant difference regarding 25-hydroxyvitamin D levels in children treated or not with antiepileptics. Due to the small sample of the study and patients in the groups with and without antiepileptic drug use, these results should be interpreted with caution. 

**Table 1:** Demographic and laboratory findings of the study subjects

Variable	Categories	Case (n=65) (Mean ± S.E*) Or n (%)	Control (n=65) (Mean ± S.E^*^) Or n (%)	*P*-value
Age (yr)		5.9±0.30	6.23±0.37	0.523*
Gender	Boys	37 (56.9)	38(58.5)	0.859**
	Girls	28 (43.1)	27(41.55)	
25-hydroxyvitamin D (ng/ml)		28.03±24.2	30.00±1.94	0.583*
	Deficient	29(44.6)	12(18.5)	0.008**
	Insufficient	15(23.1)	26(40.0)	
	Sufficient	20(30.8)	26(40.0)	
	Toxicity	1 (1.5)	1(1.5)	
Age *25-hydroxyvitamin D		Case (n=65) r (*P* value)	Control (n=65) r (*P* value)	
		-0.333(0.007***)	-0.239 (0.55***)	
Gender * 25-hydroxyvitamin D	Categories	Case (n=65) Mean ± S.E* 25-hydroxyvitamin D levels	Control(n=65) Mean ± S.E* 25-hydroxyvitamin D levels	
	Boys	30.69 ± 3.36	29.45 ± 3.31	0.506 †
	Girls	24.52 ± 3.86	30.78 ± 3.93	
	*P*-value	0.491†		0.303 †

* Standard of Error

*** Pearson Correlation Test

* Independent-Samples t-test

† Two-Way Anova

** Pearson Chi-Square Test

‡ Level of significance is considered to be <0.05

**Table 2:** 25-hydroxyvitamin D levels between CP types, GMFCS groups, and Antiepileptic usage status

Variable	Categories	n (%)	Mean ± S.E 25-hydroxyvitamin D levels			*P*-value
Type of CP, n (%)	Hemiplegic	14 (21.5)	21.34 ± 5.29			0.220**
	Quadriplegic	28 (43.1)	35.02 ± 5.90			
	Diplegic	23 (35.1)	23.60 ± 2.70			
Type of CP, n (%)	Spastic	48 (73.8)	24.84 ± 2.70			0.389**
	Hypotonic	10 (15.4)	36.42 ± 6.76			
	Dyskinetic	2 (3.1)	25.12 ± 3.38			
	Mixed	5 (7.7)	43.08 ± 27.06			
GMFCS, n (%)	I,II,III	56 (86.2)	28.61± 24.09			
	IV,V	9 (13.8)	24.33 ± 20.87			0.174**
Antiepileptic use	Yes	19 (29.2)	26.78 ± 4.11			0.791***
	No	46 (70.8)	28.55 ± 3.92			
GMFCS Categories	Categories	25-hydroxyvitamin D				
		Deficient	Insufficient	Sufficient	Toxicity	
	I- II- III	24 (42.85)	14 (25%)	17 (30.35)	1 (1.7)	0.664†
	IV- V	5 (55.55)	1 (11.11%)	3 (33.33)	0 (0)	

* Standard of Error.

† Exact Test.

** Kruskal Wallis Test.

‡ Level of significance is considered to be <0.05.

*** Independent-Samples t-test.

25-hydroxyvitamin D insufficiency is more prevalent among girls (22). 25-hydroxyvitamin D deficiency is higher in girls than boys in normal population are (15). However, in this study, although girls had lower 25-hydroxyvitamin D levels, the difference between boys and girls was not significant. In our region, the girls are dressed as most parts are covered and so have less sun exposure, which could be a cause for this finding. 

We also found that with increase in the subjects’ age, the 25-hydroxyvitamin D levels are reduced and are more prone to 25-hydroxyvitamin D deficiency. Similarly, the frequency of 25-hydroxyvitamin D deficiency was increased with age (15). Lower outdoor activity and consequently lower sun exposure are regarded as one of the causes for higher 25-hydroxyvitamin D deficiency rate in older age (23). 

We also found no correlation between significant difference in 25-hydroxyvitamin D levels among spastic, dyskinetic, mixed and hypotonic CP as well as quadriplegic, hemiplegic and diplegic types. In addition, there was no significant difference between GMFCS level and 25-hydroxyvitamin D levels. GMFCS was not associated with 25-hydroxyvitamin D levels (8). However, children in GMFCS level I to II have less severe bone deficits than children in GMFCS level III to IV (24). Most cases in our study were in GMFCS level II and III and levels I, IV and V constituted only 12 cases, which may be reason for not finding significant association. 

Some limitation of our study should be considered as follows: The large sample for GMFCS II and III was the main cause limited our analysis. The data with regard to degree of sun exposure could not be objectively assessed. The study was done on small sample of patients and in an area with different climates and cultures. 

**In conclusion, **prevalence of 25-hydroxyvitamin D deficiency was higher in this study population in comparison with healthy control children. There was significant negative correlation between age and 25-hydroxyvitamin D levels Routine measurement of 25-hydroxyvitamin D levels and proper treatment if needed, is recommended to prevent 25-hydroxyvitamin D deficiency and subsequent consequences. Future research should focus on the benefits of 25-hydroxyvitamin D supplementation for this population.
